# The mathematical model of concentration polarization coefficient in membrane transport and volume flows

**DOI:** 10.1007/s10867-016-9432-5

**Published:** 2016-11-12

**Authors:** Arkadiusz Bryll, Andrzej Ślęzak

**Affiliations:** Department of Public Health, Częstochowa University of Technology, 36b Armia Krajowa Al., 42200 Częstochowa, Poland

**Keywords:** Membrane transport, Kedem–Katchalsky equations, Concentration polarization, Concentration Rayleigh number

## Abstract

In this paper, the authors investigate the membrane transport of aqueous non-electrolyte solutions in a single-membrane system with the membrane mounted horizontally. The purpose of the research is to analyze the influence of volume flows on the process of forming concentration boundary layers (CBLs). A mathematical model is provided to calculate dependences of a concentration polarization coefficient (*ζ*_*s*_) on a volume flux (*J*_*vm*_), an osmotic force (Δ*π*) and a hydrostatic force (Δ*P*) of different values. Property *ζ*_*s*_ = *f*(*J*_*vm*_) for *J*_*vm*_ > 0 and for *J*_*vm*_ ≈ 0 and property *ζ*_*s*_ = *f*(Δ*C*_1_) are calculated. Moreover, results of a simultaneous influence of Δ*P* and Δ*π* on a value of coefficient *ζ*_*s*_ when *J*_*vm*_ = 0 and *J*_*vm*_ ≠ 0 are investigated and a graphical representation of the dependences obtained in the research is provided. Also, mathematical relationships between the coefficient *ζ*_*s*_ and a concentration Rayleigh number (*R*_*C*_) were studied providing a relevant graphical representation. In an experimental test, aqueous solutions of glucose and ethanol were used.

## Introduction

Cognitive and applicative research in membrane transport is carried out in different fields of science, technology, and medicine [1–4]. The possibility of the application of membranes depends on their structure, physicochemical properties, and transport properties [2, 5]. To interpret membrane transport, models provided under non-equilibrium thermodynamics [6, 7] and network thermodynamics [8, 9] are the most frequently used instruments. The Kedem-Katchalsky equations [10] are the most important research tools for the transport of solutions with different compositions and physicochemical properties throughout simple and complex membranes; this transport is generated by thermodynamic forces caused by single or complex physical fields (e.g., concentrations, pressures, temperatures). For non-electrolyte solutions, the K-K equations describe volume transport and transport of dissolved substances (solutes) involving the transport parameters of membranes, i.e., the hydraulic permeability coefficient (*L*_*p*_), the reflection coefficient (*σ*_*m*_) and the diffusive permeability coefficient (*ω*_*m*_). Usefulness of the classical as well as a modified form of the Kedem–Katchalsky equations has been confirmed repeatedly [7, 11].

The classical form of K-K equations is applicable in the study of membrane transport in homogenous solutions. Under particular existent conditions, it is assumed that the homogeneity of solutions is reached only for the initial state (*t* = 0). For *t* > 0, the homogeneity of solutions separated by the membrane is disturbed by the formation of diffusive layers, known as concentration boundary layers (CBLs) near the membrane [12–14]. The layers reduce the concentration gradient across the membrane, causing a decrease of the volume flows of solution and solute [15]. The reason for the formation of CBLs is the membrane itself being a natural barrier by the volume flows and the solute flows. The flows are affected by the type of membrane (its size and shape of pores that may block the flow of solute particles or may cause the retention of solute particles inside the membrane) as well as the type of solute. Therefore, there is a need to characterize the CBL layers that constitute pseudo-membranes and have an impact on the flows discussed above. To extend the range of application of the K-K equations, some modifications are made in the classical form of the K-K equations as well as in their network form developed by Peusner [15–19].

A detailed study of the phenomenon of concentration polarization is important for technical and medical issues. In technology, study results may help to develop membrane filtration or water purification in wastewater treatment plants, however, instead of solid membranes very often liquid membranes are applied. As far as medicine is concerned, it is crucial to evaluate the amount of nutrients and medicines flowing into cells throughout the cell membranes as well as the amount of unneeded substances flowing out of the cells. Membrane cells are organic membranes and therefore specialists on cellular transport should take into account that some amounts of substance might not reach inside cells due to the phenomenon of concentration polarization. Similarly, it may happen in the event of ulcer treatment by applying membranes. Considering barriers in the form of concentration layers, it should be evaluated carefully how much medicine provided to a wound actually reaches the wound.

One way to evaluate the influence of concentration polarization on membrane transport is to derive and calculate the coefficients *ζ*_*p*_, *ζ*_v_, *ζ*_*s*_ and *ζ*_*a*_ appearing in Eqs. (1) and (2). The numeric value of the coefficients indicates how strong the influence of the concentration boundary layers on membrane transport is. In previous research, the problem of the role of volume flows generated by osmotic forces (Δ*π*) and hydrostatic forces (Δ*P*) in forming concentration boundary layers was mentioned [27]. To develop this issue, we will study how the volume flux (*J*_*v*_), the osmotic force (Δ*π*) and the hydrostatic force (Δ*P*) influence the value of coefficient *ζ*_*s*_. This paper presents two mathematical models: the former presenting the influence of the volume flux (*J*_*v*_) on the value of coefficient *ζ*_*s*_ and the latter presenting the influence of the osmotic force (Δ*π*) and the hydrostatic force (Δ*P*) on the value of coefficient *ζ*_*s*_.

## Theory

The classical K-K equations for transport generated by osmotic pressure difference (Δ*π*) and hydrostatic pressure difference (Δ*P*) through the membrane describe the volume flux (*J*_*v*_) and the solute flux (*J*_*s*_) in the following form:

$$ \begin{array}{l}{J}_v={L}_p\left(\varDelta P-{\sigma}_m\right)\hfill \\ {}{J}_s=\omega \varDelta \pi +\overline{C}\left(1-{\sigma}_m\right){J}_v\hfill \end{array} $$

where *J*_*v*_ and *J*_*s*_ are volume and solute fluxes, respectively; *L*_*p*_, *σ*_*m*_ and *ω*_*m*_ are coefficients of hydraulic permeability, reflection and solute permeability, respectively; Δ*P* = *P*_*h*_ − *P*_*l*_ is the difference of hydrostatic pressure (*P*_*h*_ and *P*_*l*_ denote the higher and lower values of hydrostatic pressure, respectively); *Δπ* = *RT*(*C*_*h*_ − *C*_*l*_) is the difference of osmotic pressure (*RT* means the product of the gas constant and thermodynamic temperature, *C*_*h*_ is the solution concentration in the higher compartment of the membrane system and *C*_*l*_ is the solution concentration in the lower compartment of the membrane system). $\overline{C}=\left({C}_h-{C}_l\right){\left[ \ln \left({C}_h{C_l}^{-1}\right)\right]}^{-1}\approx 0,5\left({C}_h+{C}_l\right)=$ the average mean solute concentration in the membrane system.

The phenomenological coefficients *L*_*p*_ ,*σ*_*m*_, *ω*_*m*_ have the following interpretation:$$ \begin{array}{l}{L}_p={\left(\frac{J_v}{\varDelta P}\right)}_{\varDelta \pi =0},\hfill \\ {}{\sigma}_m={\left(\frac{\varDelta P}{\varDelta \pi}\right)}_{J_v=0},\hfill \\ {}{\omega}_m={\left(\frac{J{}_s}{\varDelta \pi}\right)}_{J_v}=0,\hfill \end{array} $$ where: [*J*_*v*_] = *m*⋅*s* ^−1^, [*J*_*s*_] = *mol*⋅*s* ^−1^⋅*m* ^−2^, [Δ*P*] = [Δ*π*] = *N*⋅*m* ^−2^ = *Pa*, [*L*_*p*_] = *m* ^3^⋅*N* ^−1^⋅*s* ^−1^, [$\overline{C}$] = *mol*⋅ *m* ^−3^, [*ω*_*m*_] = *mol*⋅*N* ^−1^⋅s^−1^, *σ*_*m*_ − the dimensionless coefficient.

It should be pointed out that it is possible to derive numeric values of coefficients *L*_*p*_, *σ* and *ω* in a series of independent tests [6].

Under conditions of concentration polarization in membrane flows, the K-K equations are modified [17]^1^:

$$ \begin{array}{l}{J}_{vs}={L}_{ps}\left(\varDelta P-{\sigma}_s\varDelta \pi \right)\hfill \\ {}{J}_{ss}={\omega}_s\varDelta \pi +\overline{C}\left(1-{\sigma}_{sa}\right){J}_{vs}\hfill \end{array} $$

Applying particular coefficients of concentration polarization, namely hydraulic *ζ*_*p*_ = *L*_*ps*_/*L*_*p*_, osmotic *ζ*_*v*_ = *σ*_*s*_/*σ*_*m*_, diffusive *ζ*_*s*_ = *ω*_*s*_/*ω*_*m*_ and advective *ζ*_*a*_ = *σ*_*sa*_/*σ*_*m*_, the above equations take the following form:

1 $$ {J}_{vs}={\zeta}_p{L}_p\left(\varDelta P-{\zeta}_v{\sigma}_m\varDelta \pi \right) $$

2 $$ {J}_{ss}={\zeta}_s{\omega}_m\varDelta \pi +\overline{C}\left(1-{\zeta}_a{\sigma}_m\right){J}_{vs} $$

Taking into account Eq. (1), (2) can be written in the form:

2a $$ {J}_{ss}=\left[{\zeta}_s{\omega}_m-\overline{C}\left(1-{\zeta}_a{\sigma}_m\right){\zeta}_p{L}_p{\zeta}_v{\sigma}_m\right]\varDelta \pi +\overline{C}\left(1-{\zeta}_a{\sigma}_m\right){\zeta}_p{L}_p\varDelta P $$

where $\overline{C}$ (1 – *ζ*_*a*_ *σ*_*m*_)*ζ*_*p*_ *L*_*p*_ = *ω*_*sa*_ is the advective diffusion permeability coefficient under the concentration polarization conditions. For *ζ*_*p*_ = *ζ*_*v*_ = *ζ*_*s*_ = *ζ*_*a*_ = 1, the K-K equations take the classical form.

In homogeneous solutions (stirred mechanically), membrane transport does not depend on the orientation of the membrane in terms of the gravity direction but for non-homogenous solutions (unstirred mechanically) this dependence is obvious [13, 15, 20–23]. The papers quoted above prove that there is clear asymmetry between the volume flux and the solution flux connected with the position of the selective membrane in terms of the gravitation vector ($\overrightarrow{g}$). Also, when the density of the solution placed over the membrane is higher than the density of the solution placed under the membrane, the convection takes place in the areas of the concentration boundary layers [21–23]. For *J*_*v*_ = 0, the concentration Rayleigh number (*R*_*Cl*_ and *R*_*Ch*_) for the layers l_l_ and l_h_ may be introduced by the following equations [24]:

3 $$ {R}_{Cl}=g{\omega}_m{\zeta}_sRT\frac{\partial \rho }{\partial C}{\delta_l}^4\left({C}_h-{C}_l\right){\left({D_l}^2{\rho}_l{\nu}_l\right)}^{-1} $$

4 $$ {R}_{Ch}=g{\omega}_m{\zeta}_sRT\frac{\partial \rho }{\partial C}{\delta_h}^4\left({C}_h-{C}_l\right){\left({D_h}^2{\rho}_h{\nu}_h\right)}^{-1} $$

where *g* is acceleration due to gravity, *∂ρ*/*∂C* is the variation of density with concentration, *D*_*l*_ and *D*_*h*_ are the diffusion coefficients, *ρ*_*l*_ and *ρ*_*h*_ are the mass density and *ν*_*l*_ and *ν*_*h*_ are the kinematic viscosity.

In non-selective membranes with the concentration Rayleigh number within the range 10^10^ ≤ *R*_*C*_ ≤ 10^11^, convection cells with a ‘plum structure’ appear near the solution above the membrane [25].

**Fig. 1:**
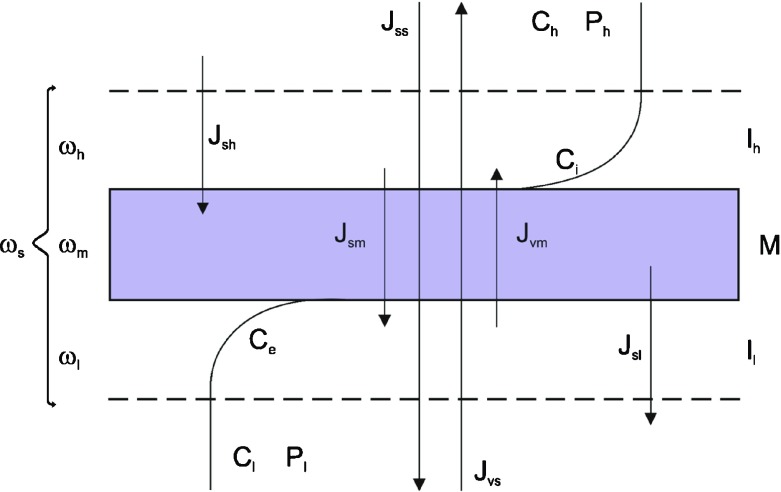
The membrane system: M − membrane; l_l_, l_h_ − concentration boundary layers (CBLs); *ω*_*s*_, *ω*_*l*_, *ω*_*m*_, *ω*_*h*_ − solute permeability coefficient; *P*_*l*_ , *P*_*h*_ − hydrostatic pressure outside the layers; *P*_*e*_, *P*_*i*_ − mechanical pressure at the boundary l_l_/M and M/l_h_; *C*_*l*_, *C*_*h*_ − solution concentrations outside the layers; *C*_*e*_, *C*_*i*_ − solution concentrations at the boundary l_l_/M and M/l_h_. Volume fluxes through the layers l_l_, l_h_, membrane M and the system l_l_/M/l_h_ are denoted by *J*_*vl*_, *J*_*vh*_, *J*_*vm*_ and *J*_*v*_; *J*_*l*_, *J*_*h*_, *J*_*m*_ and *J*_*s*_ − solute fluxes [17, 26]

Following the reasoning presented in previous papers [17, 26], let us consider the single-membrane system presented in Fig. 1, in which compartments with aqueous, non-homogeneous (unstirred mechanically) and not reacting chemically solutions of the same non-electrolyte substance are separated by the porous, symmetric, selective and electrically neutral membrane M. In this system, under isothermal conditions, water and solute, diffusing through the membrane, form the concentration boundary layers denoted by l_l_ and l_h_ at both sides of the membrane. The CBLs constitute pseudo-membranes with thicknesses of *δ*_*l*_ and *δ*_*h*_ and their transport properties are defined by the reflection coefficient of zero (*σ*_*l*_ = *σ*_*h*_ = 0) and the coefficients of non-zero of solution permeability (*ω*_*l*_, *ω*_*h*_). Let us denote solution concentrations at the boundaries l_l_/M and M/l_h_ by *C*_*e*_ and *C*_*i*_ and concentrations beyond the layers l_l_ and l_h_ respectively by *C*_*l*_ and *C*_*h*_ (*C*_*l*_ < *C*_*e*_ < *C*_*i*_ < *C*_*h*_). The mechanical pressure will be denoted by *P*_*l*_ and *P*_*h*_ (*P*_*h*_ > *P*_*l*_). For the solutions unstirred mechanically, we have Δ*π*_*m*_ = *RT*(*C*_*i*_ – *C*_*e*_).

The transport properties of the membrane are defined by the coefficients of hydraulic permeability (*L*_*p*_), reflection (*σ*_*m*_) and solute permeability (*ω*_*m*_). The reflection coefficient and the solution permeability coefficient for the system l_l_/M/l_h_ are denoted respectively by *σ*_*s*_ and *ω*_*s*_. The diffusion coefficients in layers (l_l_) and (l_h_) are denoted respectively by *D*_*l*_ and *D*_*h*_. Between the coefficients *ω*_*l*_, *ω*_*h*_, *ω*_*m*_ and *ω*_*s*_, the relation *ω*_*s*_^−1^ = *ω*_*m*_^−1^ + *ω*_*l*_^−1^ + *ω*_*h*_^−1^ appears, where *ω*_*l*_ = *D*_*l*_(*RTδ*_*l*_)^−1^, *ω*_*h*_ = *D*_*h*_(*RTδ*_*h*_)^−1^ and *RT* is the product of the gas constant and thermodynamic temperature. Definitions of the coefficients *L*_*p*_, *σ*_*m*_, *σ*_*s*_, *ω*_*l*_, *ω*_*h*_, *ω*_*m*_ and *ω*_*s*_ are provided in the paper [26]. They do not differ from the definitions given above, however, they refer to the membrane and the layers close to the membrane (upper and lower). According to Fig. 1, the solution fluxes through layers (l_l_) and (l_h_), the membrane (M) and the system l_l_/M/l_h_ are denoted by *J*_*sl*_, *J*_*sm*_, *J*_*sh*_ and *J*_*ss*,_, respectively. The volume fluxes through the elements mentioned above are denoted by *J*_*vm*_ and *J*_*vs*_. The volume flux (*J*_*vs*_) may be calculated on the basis of Eq. (1) and the volume flux (*J*_*vm*_) in the membrane system presented in Fig. 1 can be calculated on the basis of equation:

5 $$ {J}_{vm}={L}_p\left[\varDelta P-{\sigma}_mRT\left({C}_i-{C}_e\right)\right] $$

The concentration difference *C*_*i*_ and *C*_*e*_, appearing in the equation above, can be calculated for the steady state satisfying the relations:

6 $$ {J}_{vm}={J}_{vs} $$

7 $$ {J}_{sh}={J}_{sm}={J}_{sl}={J}_{ss} $$

To calculate the difference *C*_*i*_–*C*_*e*_, we use the algorithm presented in previous papers [26–28]. For the layers l_l_ and l_h_, (indexes *sl* and *sh*), the membrane (index *sm*) and the system l_l_/M/l_h_ (index *ss*) and using the K-K equations, we may write the equations:

8 $$ {J}_{sl}={D}_l{\delta_l}^{-1}\left({C}_e-{C}_l\right)+{J}_{vm}{\overline{C}}_l $$

9 $$ {J}_{sh}={D}_h{\delta_h}^{-1}\left({C}_h-{C}_i\right)+{J}_{vm}{\overline{C}}_h $$

10 $$ {J}_{ss}={\zeta}_s{\omega}_mRT\left({C}_h-{C}_l\right)+{J}_{vm}\left(1-{\zeta}_a{\sigma}_m\right){\overline{C}}_s $$

where $$ {\overline{C}}_h $$ = 0.5(*C*_*h*_ *+ C*_*i*_), $$ {\overline{C}}_l $$ = 0.5(*C*_*e*_ *+ C*_*l*_), $$ {\overline{C}}_s $$ = 0.5(*C*_*h*_ *+ C*_*l*_), 0 ≤ *ζ*_*s*_ ≤ 1 and

11 $$ {\zeta}_s={D}_l{D}_h{\left[{D}_l{D}_h+RT{\omega}_m\left({D}_h{\delta}_l+{D}_l{\delta}_h\right)\right]}^{-1} $$

Using Eq. (6) - (10), we obtain:

12 $$ {C}_i=\frac{D_h{C}_h-{\zeta}_s{\omega}_m{\delta}_h\varDelta \pi +{J}_{vm}{\delta}_h\left({\zeta}_a{\sigma}_m{\overline{C}}_s-{\scriptscriptstyle \frac{1}{2}}{C}_l\right)}{D_h-{\scriptscriptstyle \frac{1}{2}}{J}_{vm}{\delta}_h} $$

13 $$ {C}_e=\frac{D_l{C}_l+{\zeta}_s{\omega}_m{\delta}_l\varDelta \pi +{J}_{vm}{\delta}_l\left({\scriptscriptstyle \frac{1}{2}}{C}_h-{\zeta}_a{\sigma}_m{\overline{C}}_s\right)}{D_l+{\scriptscriptstyle \frac{1}{2}}{J}_{vm}{\delta}_l} $$

In the paper [29], it was proved that the coefficients *ζ*_*s*_ and *ζ*_*a*_ do not differ significantly and therefore we use only the coefficient *ζ*_*s*_. Similarly, for particular solutes the coefficients *L*_*p*_ and *L*_*ps*_ do not differ significantly, therefore we assume that *L*_*p*_ *= L*_*ps*_.

Including Eq. (12) and (13) in Eq. (5) while assuming that *ζ*_*s*_ = *ζ*_*a*_, and performing simple algebraic calculations, we obtain:

14 $$ {\zeta}_s=\frac{{J_{vm}}^3+{\varphi}_0{J_{vm}}^2+{\varphi}_1{J}_{vm}+{\varphi}_2}{\mu_0{J}_{vm}-{\mu}_1} $$

where:

- *φ*_0_ = −2[(*D*_*h*_ *δ*_*l*_ – *D*_*l*_ *δ*_*h*_) + 0.5*L*_*p*_ *δ*_*l*_ *δ*_*h*_(Δ*P* + σ_*m*_Δ*π*)](*δ*_*l*_ *δ*_*h*_)^−1^,
- *φ*_1_ = [2*L*_*p*_Δ*P*(*D*_*h*_ *δ*_*l*_ – *D*_*l*_ *δ*_*h*_) – 4*D*_*l*_ *D*_*h*_]*δ*_*l*_^−1^ *δ*_*h*_^−1^,
- *φ*_2_ = 4*L*_*p*_ *D*_*l*_ *D*_*h*_ (Δ*P* – *σ*_*m*_Δ*π*)*δ*_*l*_^−1^ *δ*_*h*_^−1^,
- *μ*_0_ = 4*L*_*p*_ *σ*_*m*_^2^ *RT* $$ \overline{C} $$(*D*_*l*_ *δ*_*h*_ + *D*_*h*_ *δ*_*l*_)*δ*_*l*_^−1^ *δ*_*h*_^−1^,
- *μ*_1_ = 4*L*_*p*_ *σ*_*m*_Δ*πω*_*m*_ *RT*(*D*_*l*_ *δ*_*h*_ + *D*_*h*_ *δ*_*l*_) *δ*_*l*_^−1^ *δ*_*h*_^−1^.

The parameters in Eq. (14) are easy to measure. In a series of independent tests we are able to derive the parameters of the membrane (*L*_*p*_, *σ*_*m*_ and *ω*_*m*_), solutions (*D*_*l*_, *D*_*h*_), volume flux (*J*_*vm*_) and thicknesses of CBL (*δ*_*l*_, *δ*_*h*_) [6, 13, 14, 24, 30, 35].

The study of coefficient *ζ*_*s*_, described in Eq. 14, is significantly important in membrane flows. The coefficient not only includes the phenomenon of the concentration polarization but also facilitates its measuring. This is essential in the event of flows through cell membranes when estimating amounts of nutrients and medicines reaching inside cells. By ignoring the concentration polarization phenomenon, we are not provided with the full and clear image of membrane flows.

**Fig. 2:**
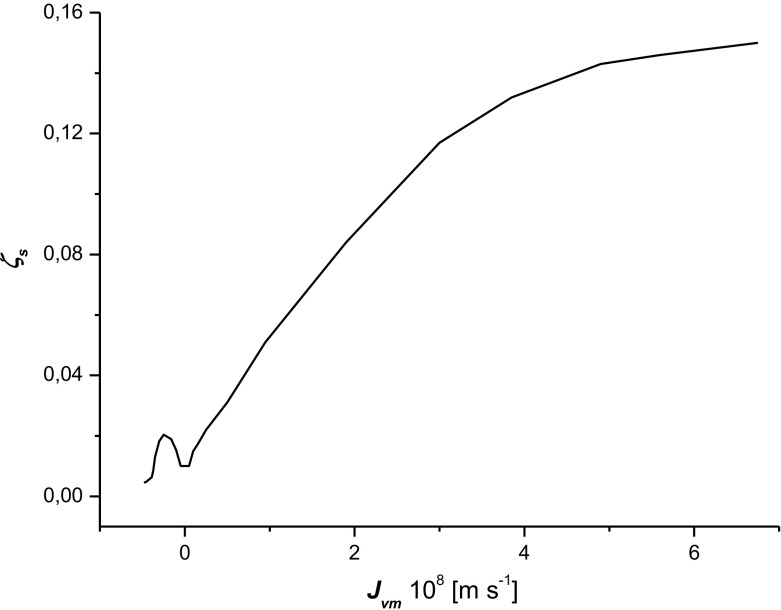
The dependence *ζ*_*s*_ = *f*(*J*_*vm*_)_Δ*P*=0_ calculated according to Eq. (14)

**Fig. 3:**
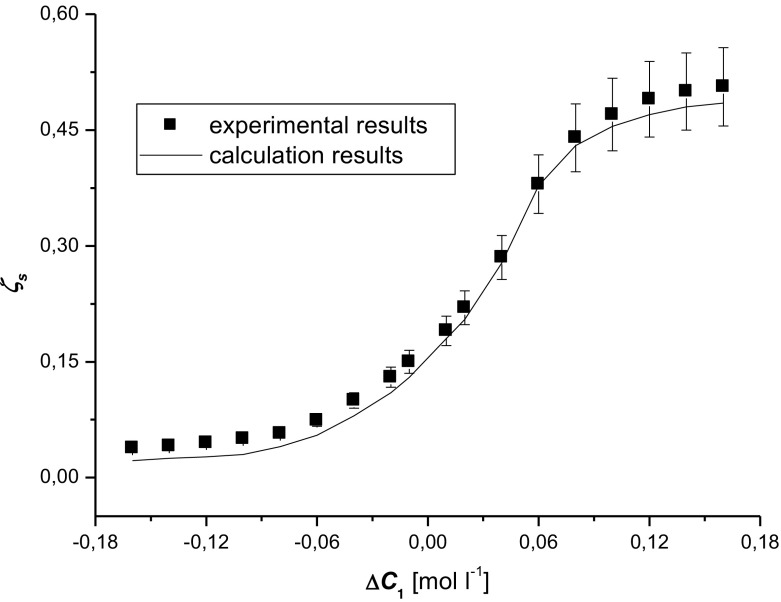
Dependence of the concentration polarization coefficient on the concentration *ζ*_*s*_ = *f*(Δ*C*_1_) for an aqueous glucose solution and Nephrophan membrane. The calculations were made according to Eq. (14). Experimental results were taken from the paper [17]

**Fig. 4:**
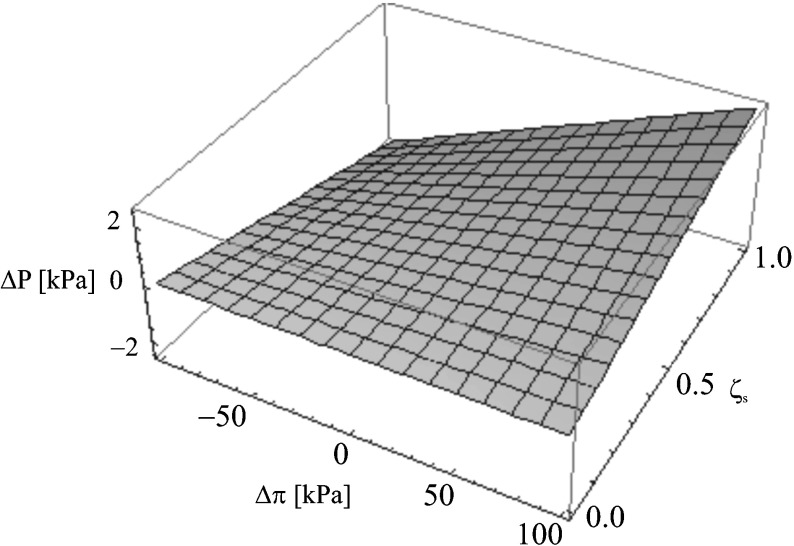
The dependence *ζ*_*s*_ = *f*(Δ*P*, Δ*π*)_*Jvm*=0_ for the aqueous ethanol solution

**Fig. 5:**
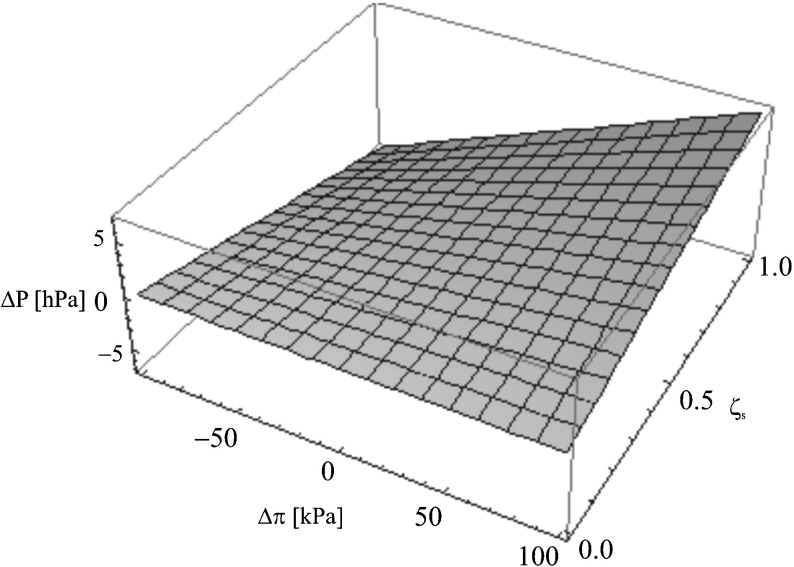
The dependence *ζ*_*s*_ = (Δ*P*, Δ*π*)_*Jvm*=0_ for the aqueous glucose solution

**Fig. 6:**
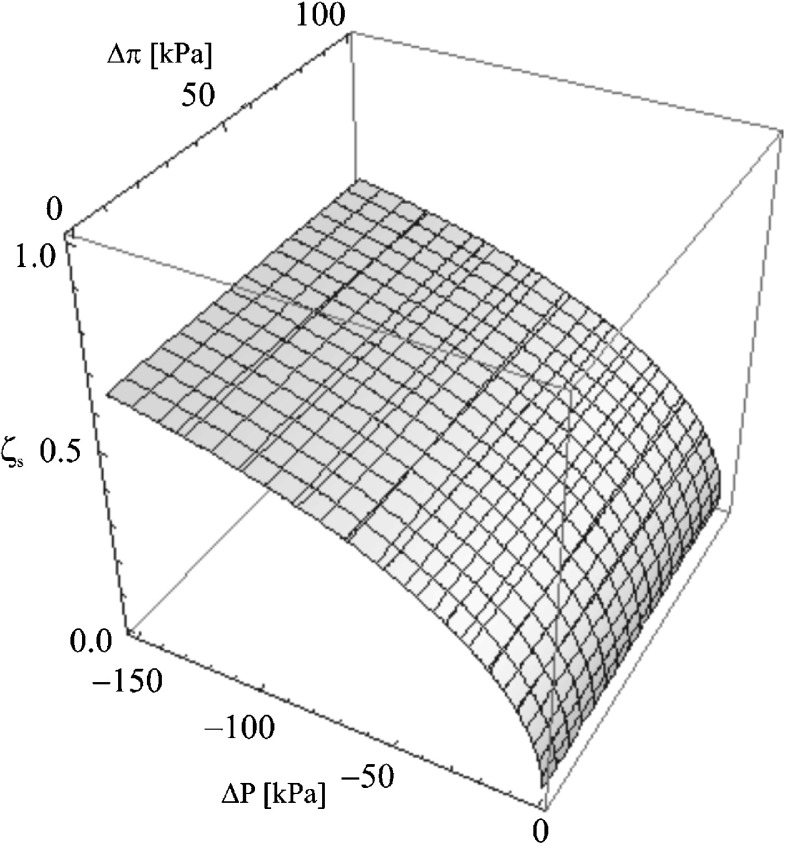
The dependence *ζ*_*s*_ = (Δ*P*, Δ*π*) dla *J*_*vm*_ ≠ 0 for the aqueous ethanol solutions

**Fig. 7:**
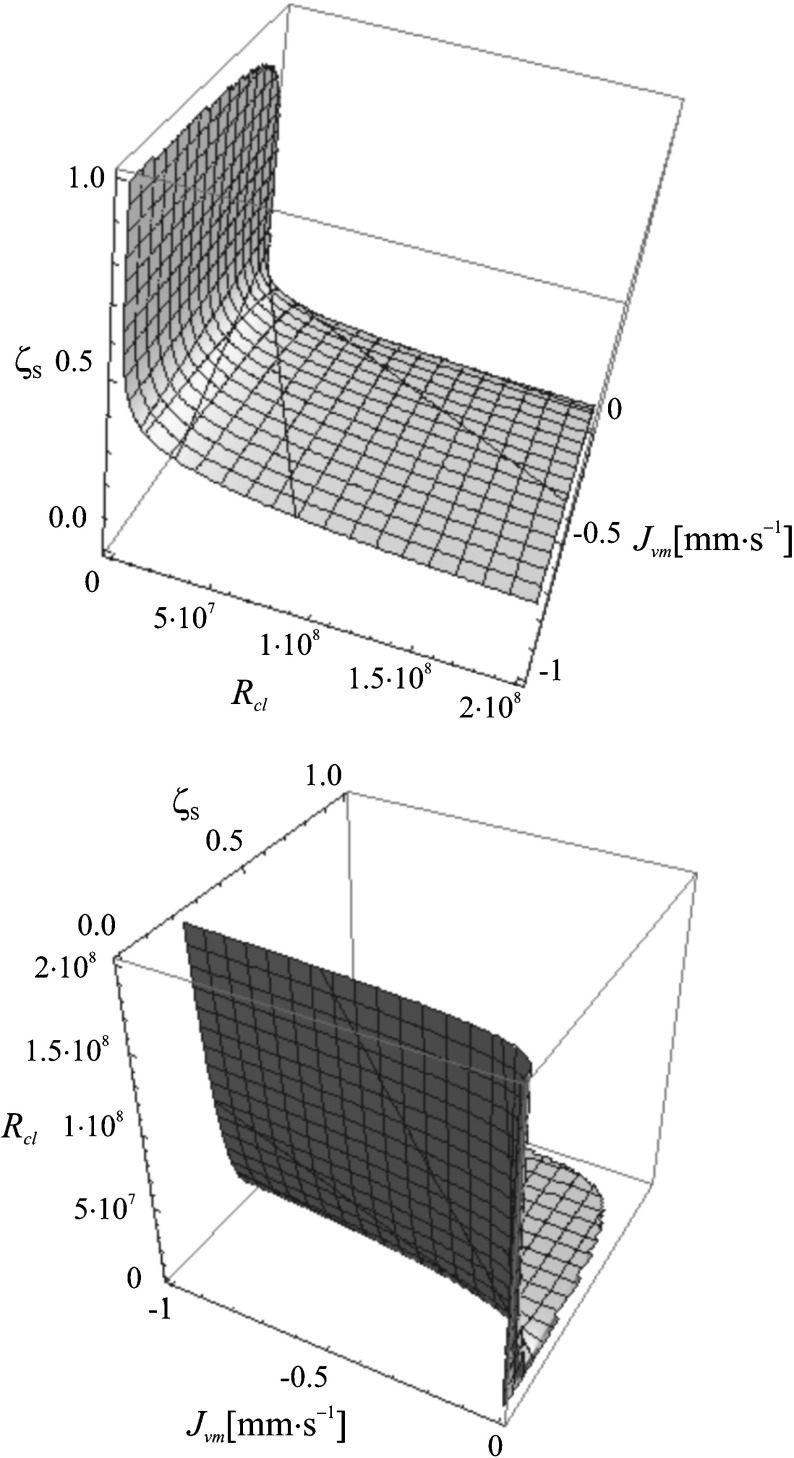
Graphical representation of dependence *ζ*_*s*_ = *f*(*R*_*Cl*,_ *J*_*vm*_) for the aqueous ethanol solution (in two projections)

We aim to prove that the detailed investigation of coefficient *ζ*_*s*_ shows its dependence on the flux *J*_*vm*_ (Fig. 2), the concentration Δ*C*_*1*_ (Fig. 3), the hydrostatic and osmotic pressure Δ*P* (Figs. 4, 5 and 6), the volume flux *J*_*vm*_ and the concentration Rayleigh number *R*_*c*_ (Fig. 7).

Let us consider the following models related to Eq. (14):

1. Assuming that *δ*_*l*_ = *δ*_*h*_ = *δ* and *D*_*l*_ = *D*_*h*_ = *D*, we obtain:

- *φ*_0_ = −*L*_*p*_(Δ*P* + *σ*_*m*_Δ*π*),
- *φ*_1_ = −4*D* ^2^ *δ* ^−2^,
- *φ*_2_ = 4*L*_*p*_ *D* ^2^ *δ* ^−2^(Δ*P* – *σ*_*m*_Δ*π*),
- *μ*_0_ = 8*L*_*p*_ *σ*_*m*_^2^ *RT* $$ \overline{C} $$ *Dδ* ^*−*1^,
- *μ*_1_ = 8*L*_*p*_ *σ*_*m*_Δ*πω*_*m*_ *RTD δ* ^−1^.

2. For *J*_*vm*_ = 0, Eq. (14) will be simplified to the following form:

15 $$ {\zeta}_s=\frac{D_l{D}_h}{\omega_mRT\left({D}_l{\delta}_h+{D}_h{\delta}_l\right)}\left(1-\frac{\varDelta P}{\sigma_m\varDelta \pi}\right) $$

In order to eliminate the volume flux (*J*_*vm*_) from Eq. (14), we use the following equation:

16 $$ {J}_{vm}={J}_{vs}={L}_p\left(\varDelta P-{\sigma}_m{\zeta}_s\varDelta \pi \right) $$

Including Eq. (16) in Eq. (14), we obtain:

17 $$ {Z}_1{\zeta_s}^3+{Z}_2{\zeta_s}^2+{Z}_3{\zeta}_s+{Z}_4=0 $$

where:

- Z_1_ = *L*_*p*_^3^ *σ*_*m*_^3^(Δ*π*)^3^,
- Z_2_ = *L*_*p*_^2^ *σ*_*m*_^2^[*L*_*p*_ *δ*_*l*_ *δ*_*h*_ (Δ*π*)^2^[*σ*_*m*_Δ*π* – 2Δ*P*] + 2(Δ*π*)^2^ (*D*_*h*_ *δ*_*l*_ – *D*_*l*_ *δ*_*h*_) – 4*RT* $$ {\overline{C}}_s $$(*D*_*h*_ *δ*_*l*_ +
- *D*_*l*_ *δ*_*h*_)*σ*_*m*_Δ*π* ]*δ*_*l*_^−1^ *δ*_*h*_^−1^
- Z_3_ = [*L*_*p*_^3^ *σ*_*m*_ *δ*_*l*_ *δ*_*h*_Δ*π*(Δ*P*)^2^ – 2(*D*_*h*_ *δ*_*l*_ – *D*_*l*_ *δ*_*h*_)*L*_*p*_^2^ *σ*_*m*_Δ*π*Δ*P* – 2*L*_*p*_^3^ *σ*_*m*_^2^ *δ*_*l*_ *δ*_*h*_Δ*P*(Δ*π*)^2^ –
- 4*D*_*l*_ *D*_*h*_ *L*_*p*_ *σ*_*m*_Δ*π* + 4*L*_*p*_^2^ *σ*_*m*_^2^ *RT* $$ {\overline{C}}_s $$(*D*_*h*_ *δ*_*l*_ + *D*_*l*_ *δ*_*h*_)Δ*P* –
- 4*L*_*p*_ *σ*_*m*_ *ω*_*m*_ *RT*(*D*_*h*_ *δ*_*l*_ + *D*_*l*_ *δ*_*h*_)Δπ]*δ*_*l*_^−1^ *δ*_*h*_^−1^
- Z_4_ = [4*L*_*p*_ *D*_*l*_ *D*_*h*_ *σ*_*m*_Δ*π* + *L*_*p*_^3^ *δ*_*l*_ *δ*_*h*_(Δ*P*)^2^ *σ*_*m*_Δ*π* ]*δ*_*l*_^−1^ *δ*_*h*_^−1^

## Calculation results and discussion

Calculations were made for the Nephrophan membrane, aqueous glucose solutions (lower index 1) and aqueous ethanol solutions (lower index 2). The coefficient of hydraulic permeability of the Nephrophan membrane for water is *L*_*p*_ = 5 × 10^−12^ m^3^N^−1^s^−1^. The values of the reflection coefficient and diffusive permeability coefficient of the membrane for the glucose and ethanol are respectively *σ*_*m*1_ = 0.068, *ω*_*m*1_ = 8 × 10^−10^ mol N^−1^s^−1^, *σ*_*m*2_ = 0.025 and *ω*_*m*2_ = 14.3 × 10^−10^ mol N^−1^s^−1^. The diffusion of each individual component in the solution is characterized by the following coefficients: *D*_1_ = 0.69 × 10^−9^ m^2^s^−1^ and *D*_2_ = 1.57 × 10^−9^ m^2^s^−1^. The volumes of the compartments (l, h) were the same and equal to 200 cm^3^. For the low glucose and ethanol concentration, we have *ρ*_*h*_ = *ρ*_*l*_(1 + *α*_1_ *C*_1*h*_ + *α*_2_ *C*_2*h*_), *ν*_*h*_ = *ν*_l_(1 + *γ*_1_ *C*_1*h*_ + *γ*_2_ *C*_2*h*_) with the coefficients *α*_1_ = *ρ*_*l*_^−1^∂*ρ*/∂*C*_1_ = 6.01 × 10^−5^ m^3^ mol^−1^, *γ*_1_ = *ν*_*l*_^−1^∂*ν*/∂*C*_1_ = 3.95 × 10^−4^ m^3^ mol^−1^, *α*_2_ = *ρ*_*l*_^−1^∂*ρ*/∂*C*_2_ = −9.02 × 10^−6^ m^3^ mol^−1^and *γ*_2_ = *ρ*_*l*_^−1^∂*ν*/∂*C*_2_ = 1.82 × 10^−5^ m^3^ mol^−1^ (*ρ*_*l*_ = 998 kg m^−3^, *ν*_*l*_ = 1.012 × 10^−6^ m^2^ s^−1^) [30]. The values *δ*_*l*_ and *δ*_*h*_ were taken from the previous paper [17]. In order to verify Eq. (14) and (17), the dependence *ζ*_*s*_ = *f*(*J*_*vm*_)_Δ*P*=0_ and *ζ*_*s*_ = *f*(Δ*C*_1_)_Δ*P*=0_ for aqueous glucose solutions was calculated. The calculation results are presented in Figs. 2, 3, and 4.

Figure 2 presents the property *ζ*_*s*_ = *f*(*J*_*vm*_)_Δ*P*=0_, i.e., the dependence of coefficient *ζ*_*s*_ on the volume flux (*J*_*vm*_) under the conditions Δ*P* = 0 (the hydrostatic fragment of the volume flux is eliminated). The property *ζ*_*s*_ = *f*(Δ*C*_1_)_Δ*P*=0,_ calculated on the basis of Eq. (14), for the aqueous glucose solutions presented in Fig. 3, has got a reverse course, i.e., it shows the monotonic change of coefficient *ζ*_*s*_ depending on the concentration Δ*C*_1_. The course of dependence shows that the value *ζ*_*s*_ in the calculations is a little bit lower than the value *ζ*_*s*_ in the test results presented in the previous paper [17], however for Δ*C*_1_ > 0, they fall into a 7% margin of measurement error.

On the basis of Eq. (17), it is possible to define the simultaneous influence of parameters Δ*π* and Δ*P* on the value of concentration polarization *ζ*_*s*_. When *J*_*vm*_ = 0, Eq. (16) proves that Δ*P = σ*_*m*_ *ζ*_*s*_Δ*π*. The value *ζ*_*s*_ is, therefore, the function of two variables Δ*P* and Δ*π* (for *σ*_*m*_ **=** const.). The function *ζ*_*s*_ *= σ*_*m*_Δ*π*Δ*P* ^−1^ is presented in the form of surface sheets in Fig. 4 (for aqueous ethanol solution) and in Fig. 5 (for aqueous glucose solution). The surface sheets are the fragments of a hyperbolic paraboloid. In the first case, Δ*π* and Δ*P* have satisfied the condition, respectively: −100 kPa ≤ Δ*π* ≤ 100 kPa and −2 kPa ≤ Δ*P* ≤ 2 kPa. In the second case, Δ*π* and Δ*P* have satisfied the condition: −100 kPa ≤ Δ*π* ≤ 100 kPa and −5 hPa ≤ Δ*P* ≤ 5 hPa.

If *J*_*vm*_ *≠* 0, then considering the dependence (16), the shape of surface *ζ*_*s*_ *=* (Δ*P*, Δ*π*) described by Eq. (14) is more complex. In the case of aqueous ethanol solution, the concentration polarization coefficient *ζ*_*s*_ is defined exclusively for the non-negative pressure Δ*π* and the non-positive pressure Δ*P*. To show the dependences *ζ*_*s*_ *=* (Δ*P*, Δ*π*), the following concentration ranges have been adopted: −150 kPa ≤ Δ*P* ≤ 0 kPa, 0 kPa ≤ Δ*π* ≤ 100 kPa. The relevant surface fragment is presented in Fig. 6. The figure and the numerical study made in *Mathematica* software proved that the value of hydrostatic pressure Δ*P* has a major influence on the value of coefficient *ζ*_*s*_. The change (variation) of the osmotic pressure value in the adopted range causes the slight change of *ζ*_*s*_.

In order to present the relation of dimensionless number *ζ*_*s*_ (the concentration polarization coefficient) with the concentration Rayleigh number (*R*_*C*_) used for describing diffusive and convective transport, for the conditions *J*_*vm*_ ≠ 0, we make some considerations using the formulas for *δ*_*l*_ and *δ*_*h*_ [31]:

18 $$ {\delta}_l={\left\{{R}_{Cl}{D}_l{\rho}_l{\nu}_l{\left[g\frac{\partial \rho }{\partial C}\left({C}_e-{C}_l\right)\right]}^{-1}\right\}}^{\frac{1}{3}} $$

19 $$ {\delta}_h={\left\{{R}_{Ch}{D}_h{\rho}_h{\nu}_h{\left[g\frac{\partial \rho }{\partial C}\left({C}_h-{C}_i\right)\right]}^{-1}\right\}}^{\frac{1}{3}} $$

Taking into account Eq. (12) and (13) in Eq. (18) and (19), after simple calculations we obtain:

20 $$ {\alpha}_1{\delta_l}^4+{\alpha}_2{\delta}_l+{\alpha}_3=0 $$

21 $$ {\beta}_1{\delta_h}^4+{\beta}_2{\delta}_h+{\beta}_3=0 $$

where:

- *α*_1_ = *g*(∂*ρ*/∂*C*){*ζ*_*s*_ *ω*_*m*_Δ*π* + *J*_*vm*_[0.5 (*C*_*h*_ – *C*_*l*_) – *ζ*_*s*_ *σ*_*m*_(*C*_*h*_ + *C*_*l*_)]}
- *α*_2_ = −0,5*J*_*vm*_ *R*_*Cl*_ *D*_*l*_ *ν*_*l*_ *ρ*_l_
- *α*_3_ = −*R*_*Cl*_ *D*_*l*_^2^ *ν*_*l*_ *ρ*_l_
- *β*_1_ = *g*(∂*ρ*/∂*C*){*ζ*_*s*_ *ω*_*m*_Δ*π* – *J*_*vm*_[0.5 (*C*_*h*_ – *C*_*l*_) + *ζ*_*s*_ *σ*_*m*_(*C*_*h*_ + *C*_*l*_)]}
- *β*_2_ = 0.5*J*_*vm*_ *R*_*Ch*_ *D*_*h*_ *ν*_*h*_ *ρ*_h_
- *β*_3_ = −*R*_*Ch*_ *D*_*h*_^2^ *ν*_*h*_ *ρ*_h_.

Let us analyze Eq. (20). Since the volume flux for Δ*P* = 0 is *J*_*vm*_ *= −L*_*p*_ *σ*_*m*_ *ζ*_*sD*_Δ*π*, consequently Δ*π* = −*J*_*vm*_(*L*_*p*_ *σ*_*m*_ *ζ*_*sD*_)^−1^. Assuming that *C*_*l*_ = 0, we have *C*_*h*_–*C*_*l*_ = *C*_*h*_ + *C*_*l*_ = *C*_*h*_ *= −J*_*vm*_(*L*_*p*_ *σ*_*m*_ *ζ*_*sD*_ *RT*)^−1^. Moreover, assuming that the CBL thickness is *δ*_*l*_ *= D*_*l*_(2*RTω*_*m*_)^−1^(*ζ*_*s*_^−1^ – 1) and *ρ*_*l*_ = *ρ*_*h*_ = *ρ*_0_, then Eq. (20) can be presented in the following form:

22 $$ {J}_{vm}{\varphi}_1{\left({\xi_s}^{-1}-1\right)}^4\left[1+\frac{J_{vm}\left({\xi_s}^{-1}-2{\sigma}_m\right)}{2RT{\omega}_m}{J}_{vm}\right]+{R}_{Cl}{\varphi}_2\left[1+\frac{1+{J}_{vm}\left({\xi_s}^{-1}-1\right)}{4RT{\omega}_m}\right]=0 $$

where: $$ {\varphi}_1=g\frac{\partial \rho }{\partial C}{D}_l^2{\left({L}_p{\sigma}_m\right)}^{-1}{(2RT)}^{-4}{\omega}_m^{-3} $$ *, φ*_2_ = *ν*_*l*_ *ρ*_o_.

The above equation presents the implicit function *ζ*_*s*_ of the variables *R*_*Cl*_ and *J*_*vm*_, with the fixed values of the remaining parameters, i.e., *ζ*_*s*_ = *f*(*R*_*Cl*_, *J*_*vm*_). The spatial graph of this function presents the dependence of concentration polarization coefficient *ζ*_*s*_ on the Rayleigh number (*R*_*Cl*_) and the volume flux (*J*_*vm*_). It is not essential to present Eq. (21) in the form of a polynomial equation of the variable *ζ*_*s*_, because we are obtaining the implicit function *ζ*_*s*_ of the variable *R*_*Ch*_ and *J*_*vm*_ anyway.

A graph of dependence *ζ*_*s*_ = *f*(*R*_*Cl*_, *J*_*vm*_) was made for the Nephrophan membrane and aqueous ethanol solution (Fig. 7). The shape of the surface in Eq. (22) proves that the concentration polarization coefficient *ζ*_*s*_ is increasing together with the decrease of the concentration Rayleigh number (*R*_*C*_) and the volume flux (*J*_*vm*_). The graph also shows the significant influence of the two parameters mentioned earlier on the value of the concentration polarization coefficient.

## Conclusions

Equations (14) and (17), derived in this paper, are useful tools for research on membrane transport under conditions of concentration polarization. Their application allows to calculate the expressions *ζ*_*s*_ = *f*(*J*_*vm*_), *ζ*_*s*_ = *f*(Δ*C*), *ζ*_*s*_ = *f*(Δ*P*, Δ*π*) and it is possible to evaluate the influence of osmotic flux (*J*_*vm*_) and/or the simultaneous operation of osmotic forces (Δ*π*) and hydrostatic forces ((Δ*P*) on the value of the concentration polarization coefficient (*ζ*_*s*_). Equations (20)-(22) and particularly (22), are very useful, too. On the basis of Eq. (22), it is easy to calculate the spatial formula *ζ*_*s*_ = *f*(*R*_*C*_, *J*_*vm*_), allowing the evaluation of the numerical relations between the concentration polarization coefficient (*ζ*_*s*_), the osmotic flux (*J*_*vm*_) and the concentration Rayleigh number (*R*_*C*_). The results of the research carried out confirmed the significant role of concentration boundary layers in osmotic and diffusive transport, in particular their applicative aspect in technology and medicine, as mentioned in the Introduction [5, 32–34]. The obtained results of the test are also significant for micro-gravitation conditions under which membrane transport and transport in areas near the membrane are of non-linear diffusive character. Under such conditions, by suppressing natural convection and/or by suppressing sedimentation, the character of the transport of oxygen and nutrients may change, thereby causing metabolism disorders [5, 31, 33].

## Symbols

*L*_*p*_: Hydraulic permeability coefficient
*J*_*v*_: Volume flux under homogeneous conditions
*J*_*vs*_: Volume flux under non-homogeneous conditions through the system l_l_/M/l_h_
*J*_*vm*_: Volume flux under non-homogeneous conditions through the membrane (M)
*σ*_*m*_: Reflection coefficient
*ω*_*m*_: Solute permeability coefficient
*ν*_*l*_, *ν*_*h*_: Kinematic viscosity of solutions in layers l_l_ and l_h_, respectively
*ρ*_*l*_, *ρ*_*h*_: Mass density of solutions in layers l_l_ and l_h_, respectively
*δ*_*l*,_*δ*_*h*_: Thickness of concentration boundary layers l_l_ and l_h_, respectively
Δ*π*: Osmotic pressure difference
Δ*P*: Hydrostatic pressure difference (Δ*P* = *P*_*h*_ – *P*_*l*_)
*P*_*h*_: *P*_*l*_, Hydrostatic pressure (*h* higher and *l* lower value)
*C*_*h*,_*C*_*l*_: Concentrations of solutions in compartments of the membrane system
*C*_*i*,_*C*_*e*_: Concentrations of solutions at boundaries l_l_/M and M/l_h_
$\overline{C}$: Mean solute concentration in the membrane
*R*: Gas constant
*R*_*C*_: Concentration Rayleigh number
*T*: Thermodynamic temperature
*D*_*l*_, *D*_*h*_: Diffusion coefficient in systems A and B
*ζ*_*p*_: Hydraulic concentration polarization coefficient
*ζ*_*v*_: Osmotic concentration polarization coefficient
*ζ*_*s*_: Diffusive concentration polarization coefficient
*ζ*_*a*_: Advective concentration polarization coefficient

## Footnotes

1 The *m* index denotes coefficients relating to the membrane, whereas the *s* index denotes coefficients relating to the system: membrane-CBLs.
